# New Issues in Life Course Research: Which Early-Life Factors Matter for Late-Life Outcomes?

**DOI:** 10.1093/geroni/igab046.101

**Published:** 2021-12-17

**Authors:** Jacqui Smith, Katrina Walsemann

## Abstract

The increased availability of retrospective information about the lives of participants in population panel studies has expanded the range of precursors to include in life course research. However, this also challenges researchers to select among many potential precursors to a late-life outcome and to determine the relative role of factors from different periods in the life course. Each paper in this symposium uses life course information from the Health and Retirement Study (HRS) to examine different late-life outcomes. Speakers will discuss what guided the particular selection of factors and outcome to examine in their study. Sonnega, Helppie-McFall, and Lee focus on indicators of childhood financial and social adversity as potential predictors of early retirement due to poor health. Park, Larkina, and Smith ask if decisions taken in early adulthood about how to balance work-and family-life by individuals and their partners are related to the categories of important life accomplishments older adults report in their life review. Two papers examine precursors of late-life health outcomes. Williams-Farrelly and Smith identified different profiles of physical activity in early- and mid-adulthood. They discuss associations between these profiles and cognitive aging. Whereas social losses, relocation, and multimorbidity are well-documented precursors of Major Depression in old age, Bergmans and Smith asked if poor health in childhood played a distal role. The session concludes with an integrative discussion of issues by Walsemann.

